# Integrated Analysis between Older Adults’ Experiences of Frailty and Korean News Media’s Frailty Discourse

**DOI:** 10.56786/PHWR.2026.19.41suppl.2

**Published:** 2026-10-29

**Authors:** Sung-Hyo Seo, Gyeong-Ye Lee, Ki-Soo Park

**Affiliations:** 1 Biomedical Research Institute, Gyeongsang National University Hospital Jinju Korea; 2 Department of Preventive Medicine, Institute of Medical Sciences, College of Medicine, Gyeongsang National University Jinju Korea

**Keywords:** Frailty, Social isolation, Healthy ageing, Social prescribing

## Abstract

**Objectives**: This study compared older adults’ frailty perceptions with Korean news media’s social discourse on frailty, examining the resulting implications for community-based frailty policies in super-aged regions.

**Methods**: Using a mixed-methods design, the study conducted focus group interviews in June 2026 with nine community-dwelling adults aged 70–78 years, and the data were analyzed thematically. Quantitatively, 369 frailty-related news articles published from 2023 to June 2026 were analyzed using Term Frequency-Inverse Document Frequency and Latent Dirichlet Allocation topic modeling. Text mining was conducted according to the actors represented, including health institutions, community-based organizations, and others. Qualitative and quantitative findings were subsequently compared and integrated.

**Results**: Older adults viewed frailty as a multidimensional, mutually reinforcing trajectory encompassing physical, cognitive, psychological, and social decline and perceived social isolation and loneliness as major drivers of frailty. News coverage involving health institutions primarily focused on sarcopenia prevention, protein and nutritional management, and disease monitoring, whereas community-oriented coverage more frequently addressed everyday support, including meal programs at senior centers. Both datasets emphasized physical function, exercise and nutrition, social isolation, and community-based prevention. Particularly, the news coverage emphasized systems and technologies, whereas older adults were increasingly concerned about independence, cognitive function, motivation, social participation, and becoming a burden on their families.

**Conclusions**: Frailty should be communicated as a condition that is preventable, delayable, and potentially improvable. Community-based programs should integrate functional assessments alongside multidomain interventions addressing physical activity, nutrition, cognitive and psychological health, and social participation.

**Key messages**

**① What is known previously?**

As a multidimensional condition, frailty stems from reduced physiological reserve and increased vulnerability to minor stressors. Exercise, adequate nutrition, and social engagement constitute key components of frailty prevention and management.

**② What new information is presented?**

Korean news coverage primarily focused on sarcopenia, nutrition and exercise, integrated care, oral health, and technology-based assessment. Conversely, older adults emphasized independence, motivation, cognitive function, social participation, and concerns about becoming a burden on their families.

**③ What are implications?**

Frailty prevention messages and community-based programs should address not only muscle strength and nutrition but also cognitive function, emotional well-being, loneliness, and social participation. Furthermore, communication on frailty should adopt clear, function-oriented language that is meaningful and accessible to older adults.

## Introduction

Frailty is a vulnerable state in which imbalance easily occurs even with minor stressors, and recovery is delayed due to a decline in multi-organ physiological reserve associated with aging [1-3]. Frailty is not identical to a disease or disability, and if detected early and managed with exercise, nutrition, chronic disease management, and social support, its progression can be slowed or function can be improved [2,3]. The World Health Organization (WHO) defines healthy aging as the process of developing and maintaining the functional ability that enables well-being in older age, and emphasizes considering not only an individual’s physical and mental intrinsic capacity but also the environment and social relationships [1].

For frailty prevention policies to be effective, the gap between the medical concepts used by experts and the meaning perceived by older adults in their daily lives must be narrowed. In previous qualitative studies, older adults associated the term “frailty” with weakness, dependency, and the final stage of life, or perceived it as a stigma, while simultaneously presenting the loss of mobility, independence, and social relationships as core experiences [4,5]. Such perceptions can influence the acceptability of frailty screening, participation in preventive behaviors, and the utilization of healthcare services.

Media coverage shapes the social agenda on the causes and solutions of frailty and is a major channel for delivering the programs of local governments and health institutions to the public. However, the extent to which the media-emphasized discourse on sarcopenia, nutrition, technology, and integrated care aligns with the independence, motivation, loneliness, and family burden that older adults actually consider important has not been sufficiently studied. Latent Dirichlet Allocation (LDA) topic modeling, which explores recurring latent topics in large-scale articles, can be utilized to identify the structure of public discourse by probabilistically estimating word distributions and topic distributions for each document [6].

Therefore, this study identified the main topics of domestic news related to frailty in older adults and the differences by implementing actors, and compared and integrated them with the perceptions of healthy aging and frailty arising from focus group interviews with community-dwelling older adults. Through this approach, we sought to propose frailty prevention messages and program design directions that can be utilized in the fields of disease control and community health promotion.

## Methods

This study applied a mixed-methods methodology combining qualitative research (focus group interviews) to explore the subjective perceptions of older adults in Jinju-si and quantitative research (big-data topic modeling) to analyze the structure of domestic frailty–related public discourse. The news articles used publicly available materials. Regarding the focus group interviews involving human subjects, approval was obtained from the Institutional Review Board of Gyeongsang National University (IRB No.: GIRB-G26-NW-0079). The purpose of the study and protection of personal information were explained to all participants, and written informed consent was obtained.

### 1. Designing and Conducting Focus Group Interviews with Older Adults

A focus group interview was conducted with nine older adults aged 70–78 years (five women and four men) in an office space suitable for interviews (a meeting room of a public institution in Jinju-si). The participants were selected from older adults in the neighborhood through recommendations from the local older adults’ association, and although all stated that they were living with their spouses, there were no married couples among the participants. The interview questions were structured primarily around the meaning of healthy aging; the difference between normal aging and frailty; physical, cognitive, psychological, and social changes that determine frailty; decline in activities of daily living; the causes and preventability of frailty; fear of frailty; and the support expected from the community and the state. In particular, open-ended questions and additional probing questions were used to comprehensively explore the participants’ perceptions of physical frailty, cognitive frailty, social frailty, and functional independence.

The interview was conducted for approximately 2 hours, from 2:00 PM to 4:00 PM, and the interview contents were recorded and transcribed with the consent of the participants. The unit of analysis for the transcribed data was a sentence representing a single, independent experience, perception, or judgment regarding aging and frailty. Everyday conversations not directly related to the research question, simple backchanneling, and repetitive expressions with no independent meaning were excluded from the analysis. Thematic analysis was conducted by categorizing statements with similar meanings and then deriving sub-themes and core themes. In the analysis process, healthy aging, physical function, cognitive and psychological changes, social relationships, preventability, and community support needs were considered as the main analysis domains.

### 2. Big Data Collection and Topic Modeling of Frailty-related Media Coverage

In order to identify the public discourse and policy approaches of Korean society regarding frailty, online news articles related to “frailty in older adults” published from January 2023 to June 2026 were collected. In a Python environment, primary URLs were collected based on the Google News Really Simple Syndication (RSS) feed, and then redirected to the original news outlet URLs using the googlenewsdecoder engine to extract the article bodies using web crawling techniques. A total of 420 articles were collected, and of these, the final 369 articles were selected for text mining analysis, excluding 51 articles that had duplicate content, promotional content, or body text collection errors.

For data cleaning, a morphological analyzer was applied to extract only common nouns and proper nouns from the body text. Subsequently, a domain-specific stopword dictionary was constructed to remove unnecessary noise words from the analysis, and the Term Frequency-Inverse Document Frequency algorithm was applied to filter out words that appeared excessively in specific articles or lacked discriminative power across the corpus, thereby establishing a refined vocabulary dictionary.

To analyze the context of the refined text data, the LDA algorithm, an unsupervised learning-based natural language processing technique, was applied. This technique mathematically reverse-calculates and extracts the proportion of latent key topics and the probability distribution of related keywords within a large document set. Furthermore, to identify differences in perspective depending on the speakers (performing agents) who produce or lead news articles, a cross-analysis was conducted by stratifying the entire dataset into three groups based on the content and subject of the articles. The three implementing entities were classified into health institutions, the community, and others. Health institutions included the Ministry of Health and Welfare, the Korea Disease Control and Prevention Agency (KDCA), public health centers and branch health centers, medical centers, the National Health Insurance Service (NHIS), and Dementia Relief Centers. The community included community service centers and administrative welfare centers, senior community centers, resident autonomy organizations, welfare centers, and village halls. Universities, research institutes, academic societies, businesses, and private organizations were classified as others.

## Results

### 1. Subjective Experience and Perception Structure of Frailty among Older Adults in Jinju

As a result of the thematic analysis of focus group interview data, the essence of frailty and perceptions of health perceived by older adults in Jinju-si were derived into a total of 10 core themes and specific sub-themes (Table 1). Older adults who participated in the interviews did not define frailty as a fragmentary state such as sarcopenia or a specific disease, but perceived it as a “complex and cascading phenomenon of functional collapse,” where physical, cognitive, mental, and social domains are organically intertwined and break down.

Notably, older adults perceive “social isolation” not only as a tragic consequence of frailty but also as a preceding cause (trigger) that first prompts frailty. Participants pointed out that psychosocial withdrawal precedes physical sarcopenia, noting, “If the mind ages first and one avoids people, the body also ages quickly” and “staying only at home invites illness.”

This finding suggests that the internal variables of “depression” and “isolation,” which were overshadowed by the physical measurements of K-FRAIL (Fatigue, Resistance, Ambulation, Illness, Loss of weight), a clinical indicator for assessing frailty, can operate much more powerfully in the actual lives of older adults. In addition, older adults perceived losing their independence in life and “becoming a burden to their families and being confined to a nursing hospital” as a greater fear than death or poverty. To avoid these events, they strongly demanded that the community provide social networks that allow many people to socialize, such as senior employment programs and communal meals at senior community centers.

### 2. Structure of Frailty Discourse in Domestic Media Reports and Differences in Perspectives by Stakeholder

As a result of LDA topic modeling on all 369 news articles, a total of six major discourses were extracted (Table 2). The first topic (T1) was interpreted as “sarcopenia prevention and nutrition- and exercise-based management in later life,” centering on words such as exercise, muscle, protein, intake, and sarcopenia. The second topic (T2) showed high weights for care, oral, medical, integration, dental, nursing care, and visiting, and was named “community-embedded integrated care alongside home-centered medical and oral health services.” The third topic (T3) addressed managing the pace of aging, chronic diseases, and lifestyle factors, focusing on aging, age, disease, diabetes, blood sugar, stress, and lifestyle. The fourth topic (T4) reflected health risks among older adults living or eating alone, with meals, blood pressure, eating alone, being alone, depressive symptoms, and decline in physical function appearing together. The fifth topic (T5) was composed of function, assessment, development, technology, clinical, program, and measurement, representing assessment of physical function and technology-based interventions. The sixth topic (T6) combined aging, prevention, program, public health, cohort, pilot, and region, and was interpreted as prevention and cohort programs based in public health centers.

Media coverage entities were classified into health institutions, community, and others. In articles related to health institutions, late-life functional decline and muscle and nutrition management, chronic disease prevention programs centered on local governments and public health centers, and home-visit medical and oral care and integrated care systems emerged as major topics. In community-related articles, closely integrated daily care—such as communal meals at senior community centers, dietary support for older adults living alone, and oral and nutritional home-visit care—and village-centered integrated care networks were emphasized. In other articles, the clinical risks of sarcopenia and oral frailty, cellular aging and lifestyles such as eating alone, and digital healthcare and assessment technologies for older adults were the main topics. Oral health, integrated care, physical function, and nutrition/meals were commonly addressed by the three executing entities; however, health institutions approached them from the perspective of systems and programs, the community from daily life support, and others from the perspective of mechanisms, evaluation, and technology.

The two data sources, news and older adults’ perceptions, converged on physical function and mobility, exercise and nutrition, living alone, eating alone, and social isolation, and the importance of community-based prevention and care (Table 3). In particular, the news topic of “health risks of living alone and eating alone” was linked in the interviews with the perception of a vicious cycle of loneliness-reduced activity-depression-frailty. In addition, the prevention and integrated care topics of articles from health institutions and the community aligned with the communal dining, exercise, resident meetings, and social participation support requested by older adults.

On the other hand, the news presented an expanded range of expert-, system-, and technology-centered elements, including oral health, chronic diseases, physical function measurement, cohorts, and digital healthcare. Older adults emphasized independence, motivation and confidence, changes in cognitive function, and the fear of becoming a burden to their families, which were relatively less prominent in the news. In other words, while media discourse described frailty as a risk state that must be measured and managed, older adults tended to describe it as an experience of losing daily roles and relationships.

## Discussion

This study analyzed domestic news discourse on frailty and the subjective perceptions of community-dwelling older adults together to identify commonalities and gaps. In the news, sarcopenia, exercise and protein, integrated care, oral health, and functional assessment and technology were the main agenda items. Older adults also viewed movement, muscle strength, exercise, and nutrition as important, but they placed the criteria for health on independent living and maintaining social relationships, and understood frailty as a process wherein physical, cognitive, emotional, and social functions mutually deteriorate. This finding aligned with the WHO’s perspective that healthy aging is a process of maintaining functional ability through the interaction between an individual’s intrinsic capacity and the environment [1].

In the perceptions of older adults, walking and outdoor activities were concrete and observable signs for judging frailty. This finding was consistent with the existing concept that slow gait and decreased muscle strength are core components of physical frailty [2,3]. However, the interview participants did not view gait decline as a mere physical problem but described it as a starting point leading to decreased self-confidence, reduced outings, and disconnection of relationships. Therefore, in public awareness campaigns, rather than emphasizing only “the state of decreasing muscle,” presenting “changes such as a slowing walking speed, reducing daily activities, and a decrease in meeting people” together may better align with the experiences of older adults.

Loneliness and social isolation were common and important issues in both the news and interviews. Longitudinal studies have reported that loneliness may be associated with the risk of subsequent progression to physical frailty [7]. In these interviews as well, loneliness was perceived as a bidirectional relationship wherein it promotes frailty through reduced activity and depression, and functional decline, in turn, makes it difficult to meet people. Rather than consisting solely of exercise classes, community-based frailty prevention programs need to incorporate social elements, such as activating small-group gatherings and connecting friends or health leaders.

While older adults accepted frailty as a frightening term, they believed it could be delayed through their own efforts. International qualitative studies have also reported that older adults’ understanding of frailty differs from medical definitions and can be linked to dependency and stigma, making the method of explanation and choice of terminology important [4,5]. Prevention messages should avoid the fatalism of “frailty that everyone experiences as they age” and inform about early signs distinguished from normal aging, modifiable factors, and community services wherein they can actually participate. Exercise can improve muscle strength, gait, balance, and physical performance in pre-frail and frail older adults [8], and interventions providing both exercise and nutrition also have the potential to help improve frailty-related indicators [9].

Differences across the performing actors of news provide implications for the design of community delivery systems. Health institutions were shown to play a role in providing the institutional foundation for prevention programs and integrated care, the community in providing communal dining at senior community centers and daily care, and the academic and technological sectors in providing assessment tools and digital technology. What older adults demanded was not encountering these elements as separate programs, but experiencing them as connected services within their daily living sphere. Public health centers can serve as a hub that conducts simple frailty screening and functional assessments and, based on the results, links exercise, nutrition, oral health, cognitive, mental health, and social participation services with community welfare programs.

This study has limitations. First, Google News RSS-based data is influenced by search algorithms and media outlets’ body text accessibility, and thus may not represent the entirety of domestic news coverage. Second, as the focus group interviews targeted nine individuals in a single urban area, it is difficult to generalize the findings to diverse older adult populations; furthermore, because all participants were older adults living as couples, their opinions on social issues such as living alone may be limited. Nevertheless, this study is significant in that it integrated large-scale news discourse with the lifeworld perspective of older adults to present core areas that frailty communication and community programs should address together.

In conclusion, domestic frailty–related news and older adults’ perceptions aligned regarding the importance of physical function, exercise and nutrition, social isolation, and community-based prevention. While the news emphasized systems and technology, older adults placed more emphasis on individual-level health status—such as physical (importance of independence), cognitive, and social activities—and concerns about the increasing burden of care on their families. Therefore, public health messages should describe frailty as an individual health status that is distinguishable from normal aging and can be prevented and improved multidimensionally (physical, nutrition, social activity, etc.), and community programs must integrate exercise, nutrition, oral health, mental health, and social connectedness along with functional assessment (Figure 1).

## Figures and Tables

**Figure 1:**
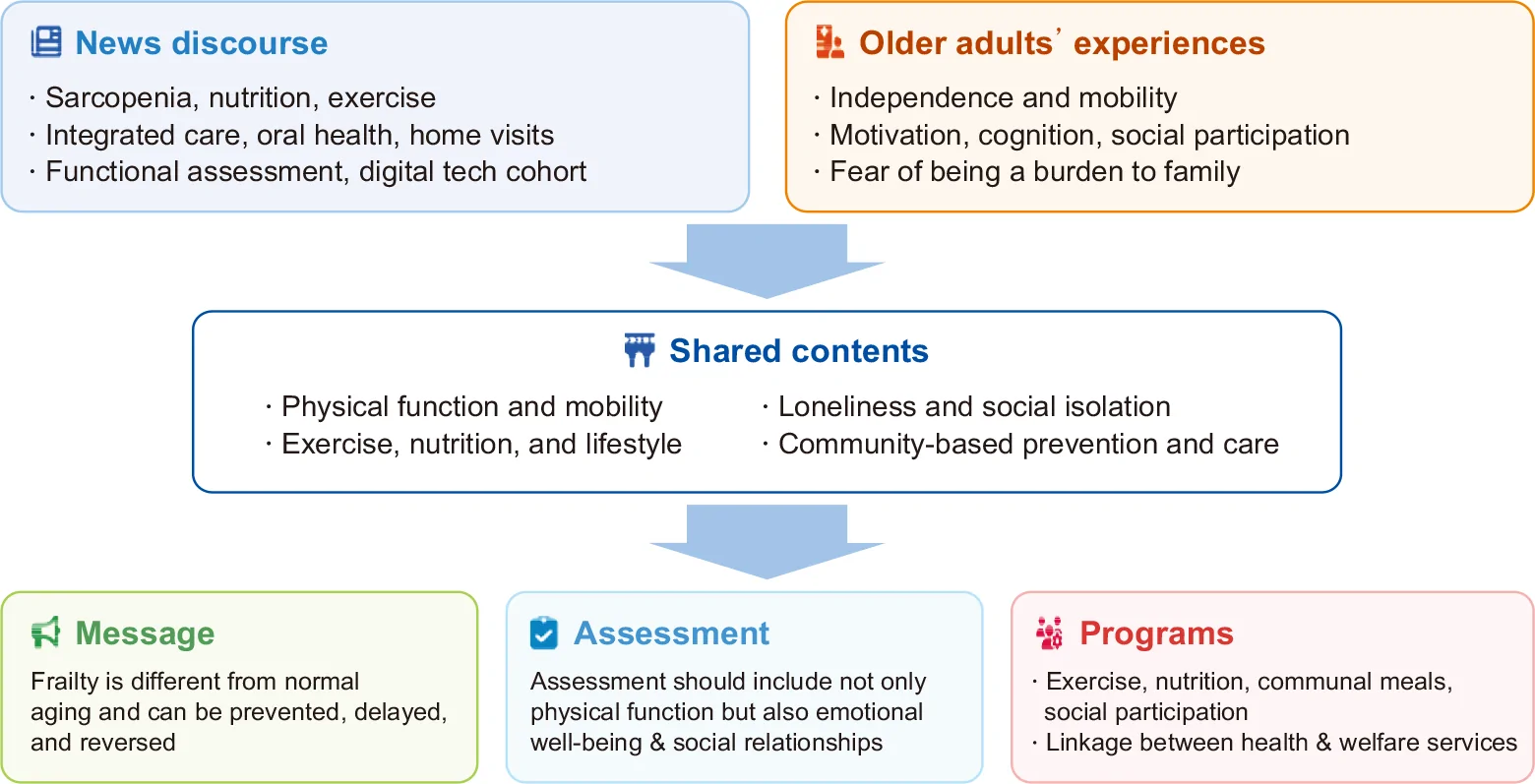
Integrating media discourse and older adults’ frailty perceptions: practical implications for community-based frailty prevention

**Table 1:** Themes related to frailty identified through focus group interviews with older adults

Theme	Sub-themes and key findings
1. Healthy aging is understood as the ability to move independently and live in a community, rather than merely being disease free.	• Independence and activity were considered central components of health, including unrestricted mobility and maintaining physical independence. • Social networks were perceived as important for sustaining health, particularly meeting friends and participating in community-based work. • Social isolation at home was considered unhealthy even in the absence of disease.
2. Frailty is not simply a consequence of aging but a process in which functional capacity and motivation deteriorate concurrently.	• Frailty was distinguished from normal aging as a state of functional decline associated with entering the final stage of life. • Early manifestations of frailty included physical weakness reflected through slower walking speed, loss of muscle mass, and chronic fatigue. • Subsequent manifestations involved psychological deterioration, including diminished motivation, reduced self-confidence, and depressed mood.
3. Mobility and social participation represent key markers distinguishing healthy aging from frailty.	• Walking speed, posture, and balance were regarded as intuitive indicators of frailty in older adults. • Healthy older adults were described as those who remained physically active and engaged socially, whereas frail older adults were those who stayed at home and avoided social contact. • Participants perceived psychological apathy as a factor that could precede and accelerate decline in physical functions.
4. Frailty is experienced as simultaneous deterioration in both physical and cognitive functions.	• Physical changes included difficulty climbing stairs or lifting objects, joint pains, and markedly increased fatigue. • Cognitive changes involved forgetting appointments, difficulty recalling words, and difficulty navigating familiar surroundings.
5. Frailty is a vicious cycle arising from the interaction between physical and psychological decline.	• The cycle consisted of declining physical function, diminished confidence and motivation, avoidance of new challenges, and reduced social activity and outings, which contributed to further physical deterioration.
6. Social isolation is perceived as a major driver of frailty.	• Loneliness, loss of friends, and lack of social interaction were regarded as factors that accelerate aging. • Participants expressed strong concerns about being confined alone at their homes.
7. Frailty can be substantially delayed through individual efforts and modifiable behaviors.	• Although frailty was not considered completely avoidable, participants believed that its progression could be substantially hindered through regular walking, adequate protein intake, positive thinking, and continued learning.
8. Loneliness and unhealthy lifestyle behaviors are key determinants of frailty.	• Seven factors were perceived as major causes of frailty: loneliness, physical inactivity, inadequate nutrition, chronic disease, aging, insufficient sleep, and lack of positive thinking. • Participants particularly emphasized the pathway in which social isolation contributes to depression and subsequently to frailty.
9. The greatest fear is becoming a family burden rather than experiencing financial hardship.	• Participants expressed considerable fear of dementia, loss of independence, and admission to a long-term care facility. • They were more concerned about imposing caregiving responsibilities on their children than experiencing financial hardships themselves.
10. Frailty prevention should be addressed through community-based efforts rather than isolated individual efforts.	• Participants requested expanded employment opportunities for older adults and community-based shared meal programs. • They also emphasized the need for infrastructure designed to support group exercise, resident gatherings, and opportunities for social participation.

**Table 2:** Main topics identified in the news corpus

Category	Topic	Representative keywords	Interpretation
T1	Sarcopenia prevention and nutrition- and exercise-based management in later life	Exercise, muscle, protein, intake, sarcopenia	Exercise and nutritional strategies to prevent muscle loss as well as physical and functional deterioration
T2	Community-embedded integrated care alongside home-centered medical and oral health services	Care, oral health, medical care, integration, long-term care, home visit	Integrated care connecting home-centered medical care, oral health management, and long-term care services
T3	Managing the pace of aging, chronic diseases, and lifestyle factors	Aging, age, diabetes, blood glucose, stress	Managing the pace of aging through chronic disease control and lifestyle modifications
T4	Health risks among older adults living or eating alone	Meals, blood pressure, eating alone, living alone, depressive symptoms	Physical and mental health risks associated with social isolation
T5	Assessment of physical function and technology-based interventions	Function, assessment, development, technology, clinical practice, measurement	Assessment tools and clinical or digital interventions
T6	Prevention and cohort programs based in public health centers	Aging, prevention, program, public health, cohort, community	Local government pilot programs and monitoring systems
Public health institutions	Prevention programs, integrated care, and home-based medical services	Prevention, program, disease, care, medical services, oral health	Institutional public health and healthcare services
Community organizations	Shared meals, daily-life support, and community networks	Meals, senior centers, living alone, oral health, care	Community-based welfare and social support integrated into daily life
Other organizations	Biological aging, sarcopenia, and digital assessment	Cells, function, technology, assessment, sarcopenia, blood pressure	Academic investigation of aging mechanisms and technology development

**Table 3:** Integrated comparison of news discourse and older adults’ perceptions

Integrated domain	News discourse	Older adults’ perceptions	Association	Public health implications
Physical function and mobility	Sarcopenia, functional assessment, and clinical interventions	Walking speed, climbing stairs, lifting objects, and going outside	Convergence	Explain walking ability, muscle strength, and daily functioning using simple and accessible language
Exercise, nutrition, and lifestyle	Protein intake, exercise, and blood glucose and diabetes management	Walking, protein intake, daily routines, and positive attitude	Convergence	Implement multidomain lifestyle interventions with repeated assessments
Social isolation	Living and eating alone, depressive symptoms, and health risks	Vicious cycle of loneliness, reduced activity, depression, and frailty	Convergence and extension	Include shared meals, social gatherings, older adults’ employment opportunities, and social prescribing
Community-based care	Public health-center programs, home-based medical and oral healthcare, and integrated care	Community exercise programs, resident gatherings, and social participation opportunities	Convergence	Strengthen connections among public health centers, welfare organizations, and community groups
Independence, cognitive function, emotional well-being, and family	Relatively limited attention	Independence, memory, motivation, and fear of becoming a burden on family members	Extension from older adults’ perspectives	Incorporate cognitive and emotional assessments and emphasize dignity and self-determination
Technology and institutional systems	Cohorts, measurement technologies, and digital healthcare	Prioritizing accessible face-to-face services over specific technologies	Difference in emphasis	Design technology to complement interpersonal relationships while improving access to services
