# Supplementary material for: Integrated Analysis between Older Adults’ Experiences of Frailty and Korean News Media’s Frailty Discourse
Source: Public Health Wkly Rep. 2026 Oct 29;19(41 Suppl):19–36. doi: 10.56786/PHWR.2026.19.41suppl.2 (PMC13725138; doi:10.56786/PHWR.2026.19.41suppl.2)
Supplement: Translation

# Translation: 노인의 노쇠 경험과 언론 보도에 나타난 노쇠 담론의 통합적 분석

**Authors:** 성효 서, 경예 이, 기수 박

**Affiliations:** 1 경상국립대학교병원 의생명연구원; 2 경상국립대학교 의과대학 의과학연구원 예방의학교실

**Keywords:** 노쇠, 사회적 고립, 건강 노화, 사회적 처방

## Abstract

**목적:**

본 연구는 초고령사회에 진입한 지역의 노인들이 주관적으로 체감하는 ‘노쇠(frailty)’에 대한 인식과 국내 언론 보도에 나타난 노쇠 관련 사회적 담론(빅데이터 토픽)을 비교 분석하여, 지역사회 노쇠 정책의 발전적 방향을 제시하고자 하였다.

**방법:**

혼합 연구 방법론(mixed methods)을 적용하여, 질적 연구로는 2026년 6월 70–78세 지역사회 거주 노인 9명을 대상으로 초점 집단 면담을 수행하고 주제 분석을 하였다. 양적 연구로는 2023년부터 2026년 6월까지 수집된 노쇠 관련 뉴스 369건을 대상으로 단어 빈도-역문서 빈도(Term Frequency-Inverse Document Frequency) 기반 어휘 선별 및 잠재 디리클레 할당(Latent Dirichlet Allocation) 토픽 모델링을 적용하여 수행 주체별(보건기관, 지역사회, 기타) 텍스트 마이닝을 수행하였다.

**결과:**

면담 참여 노인들은 노쇠를 단순한 노화가 아닌 신체‧인지‧정신‧사회적 기능이 연쇄적으로 붕괴되는 다차원적 악순환 과정으로 인식하였으며, ‘사회적 고립’과 ‘외로움’을 노쇠의 가장 결정적 원인으로 지목하였다. 반면 언론 보도 내용 분석 결과, 보건기관은 근감소증 예방, 단백질 영양 관리, 질병 모니터링 등 의료적 접근에 편중되어 있었다. 반면 커뮤니티 중심의 보도에서는 경로당 식사 지원 등 일상 밀착형 돌봄을 상대적으로 더 강조하였다. 국내 노쇠 관련 뉴스와 노인의 인식은 신체기능, 운동‧영양, 사회적 고립과 지역사회 예방의 중요성에서 일치하였지만, 뉴스는 제도와 기술을 강조한 반면 노인들은 자립성, 인지기능과 의욕, 사회참여 및 가족 부담을 더 강조하였다.

**결론:**

노쇠는 예방과 지연이 가능하며, 적절한 중재를 통해 개선될 수 있는 상태라는 점을 강조하여 인식을 개선할 필요가 있다. 지역사회 기반 노쇠 예방‧관리 프로그램은 신체기능 평가와 함께 신체활동, 영양, 인지 및 정신건강, 사회참여를 포괄하는 다영역 중재를 통합적으로 제공해야 한다.

**핵심요약**

**① 이전에 알려진 내용은?**

노쇠는 여러 생리적 체계의 예비능력이 감소하여 작은 스트레스에도 건강 상태가 크게 악화되는 다차원적 상태이며, 운동‧영양과 사회적 관계는 예방과 관리의 중요한 요소이다.

**② 새로이 알게 된 내용은?**

국내 뉴스는 근감소증, 영양‧운동, 통합돌봄, 구강건강과 기술 기반 평가를 주로 다루었으나, 노인은 자립성, 의욕, 인지기능, 사회참여와 가족에게 짐이 될 두려움을 더 중요하게 인식하였다.

**③ 시사점은?**

노쇠 예방 메시지와 지역사회 사업은 근력과 영양뿐 아니라 인지‧심리적 기능, 사회참여에 대한 노인의 요구를 반영하여야 할 것이다. 또한 노쇠에 관한 의사소통에서는 노인들이 쉽게 이해하고 자신의 삶과 연관 지어 받아들일 수 있도록, 명확하고 신체‧일상생활 기능 중심의 표현을 사용해야 한다.

## 서 론

노쇠는 노화에 따른 다기관 생리적 예비능력의 감소로 인해 작은 스트레스에도 불균형이 쉽게 발생하고 회복이 지연되는 취약한 상태이다[1-3]. 노쇠는 질병이나 장애와 동일하지 않으며, 조기에 발견하여 운동, 영양, 만성질환 관리와 사회적 지원을 제공하면 진행을 늦추거나 기능을 개선할 수 있다[2,3]. 세계보건기구는 건강 노화를 노년기의 안녕을 가능하게 하는 기능적 능력을 개발하고 유지하는 과정으로 정의하며, 개인의 신체‧정신적 내재 역량뿐 아니라 환경과 사회적 관계를 함께 고려할 것을 강조하였다[1].

노쇠 예방정책이 효과를 얻으려면 전문가가 사용하는 의학적 개념과 노인이 일상에서 체감하는 의미 사이의 간극을 줄여야 한다. 선행 질적 연구에서 노인은 ‘노쇠’라는 용어를 쇠약, 의존, 삶의 마지막 단계와 연결하거나 낙인으로 받아들이기도 하였고, 동시에 이동성‧독립성‧사회관계의 상실을 핵심 경험으로 제시하였다[4,5]. 이러한 인식은 노쇠 선별검사에 대한 수용성, 예방행동 참여, 보건의료서비스 이용에 영향을 줄 수 있다.

언론 보도는 노쇠의 원인과 해결 방안에 대한 사회적 의제를 형성하고, 지자체 및 보건기관의 사업을 대중에게 전달하는 주요 경로이다. 그러나 언론이 강조하는 근감소증, 영양, 기술 및 통합돌봄 등의 담론이 노인이 실제로 중요하게 생각하는 자립성, 의욕, 외로움 및 가족 부담과 어느 정도 일치하는지는 충분히 연구되지 않았다. 대규모 기사에서 반복되는 잠재 주제를 탐색하는 잠재 디리클레 할당(Latent Dirichlet Allocation, LDA) 토픽 모델링은 문서별 단어 분포와 주제 분포를 확률적으로 추정하여 공중 담론의 구조를 파악하는 데 활용할 수 있다[6].

이에 본 연구는 국내 노인 노쇠 관련 뉴스의 주요 주제와 수행 주체별 차이를 파악하고, 지역사회 노인의 초점 집단 면담에서 나타난 건강 노화 및 노쇠 인식과 비교‧통합하였다. 이를 통해 질병 관리 및 지역사회 건강 증진 영역에서 활용할 수 있는 노쇠 예방 메시지와 사업 구성 방향을 제안하고자 하였다.

## 방 법

본 연구는 진주시 노인의 주관적 인식을 탐색하기 위한 질적 연구(초점 집단 면담)와 국내 노쇠 관련 공적 담론 구조를 분석하기 위한 양적 연구(빅데이터 토픽 모델링)를 결합한 혼합 연구 방법론(mixed methods)을 적용하였다. 뉴스 기사는 공개된 자료를 사용하였다. 사람을 대상으로 한 초점 집단 면담에 대해서는 경상국립대학교 기관생명윤리위원회의 승인을 받았다(승인번호: GIRB-G26-NW-0079). 모든 참여자에게 연구 목적과 개인정보 보호를 설명하고 서면 동의를 받았다.

### 1. 노인 초점 집단 면담 설계 및 수행

면담하기 좋은 사무 공간(진주시 내 공공기관 회의실)에서 만 70–78세 노인 9명(여성 5명, 남성 4명)을 대상으로 초점 집단 면담을 하였다. 참여자는 지역사회 노인회의 추천을 통해 동 지역 노인으로 선정하였으며 모두 부부가 같이 살고 있다고 하였으나 대상자 중 부부는 없었다. 면담 질문은 건강한 노화의 의미, 정상 노화와 노쇠의 차이, 노쇠를 판단하는 신체적‧인지적‧심리적‧사회적 변화, 일상생활 수행능력의 저하, 노쇠의 원인과 예방 가능성, 노쇠에 대한 두려움, 지역사회 및 국가에 기대하는 지원 등을 중심으로 구성하였다. 특히 신체적 노쇠, 인지적 노쇠, 사회적 노쇠와 기능적 독립성에 대한 참여자의 인식을 포괄적으로 탐색할 수 있도록 개방형 질문과 추가 탐색 질문을 사용하였다.

면담은 오후 2시부터 4시까지 약 2시간 동안 진행하였으며, 참여자의 동의를 받아 면담 내용을 기록하고 전사하였다. 전사 자료의 분석단위는 노화와 노쇠에 대한 하나의 독립된 경험, 인식 또는 판단을 나타내는 문장으로 하였다. 연구 질문과 직접 관련이 없는 일상적 대화, 단순한 맞장구 및 독립적인 의미를 갖지 않는 반복 표현은 분석에서 제외하였다. 유사한 의미를 지닌 진술을 범주화한 후 하위 주제(sub-theme)와 핵심 주제(theme)를 도출하는 방식으로 주제 분석을 하였다. 분석 과정에서는 건강한 노화, 신체기능, 인지‧심리적 변화, 사회적 관계, 예방 가능성 및 지역사회 지원 요구를 주요 분석 영역으로 고려하였다.

### 2. 노쇠 관련 언론 보도 빅데이터 수집 및 토픽 모델링

노쇠에 대한 한국 사회의 공적 담론과 정책적 접근 방식을 파악하기 위해 2023년 1월부터 2026년 6월까지 발행된 ‘노인 노쇠’ 관련 온라인 뉴스 기사를 수집하였다. Python 환경에서 구글 뉴스(Google News) RSS (Really Simple Syndication) 피드를 기반으로 1차 웹사이트 주소를 수집한 뒤, googlenewsdecoder 엔진을 활용해 언론사 원본 웹사이트 주소로 리다이렉트(redirect)하여 기사 본문을 웹 크롤링(web crawling) 기법으로 추출하였다. 총 420건의 기사가 수집되었으며, 이 중 내용이 중복되거나 광고성 기사, 본문 수집 에러가 발생한 51건을 제외한 최종 369건을 텍스트 마이닝 분석 대상으로 확정하였다.

자료 정제를 위해 형태소 분석기를 적용하여 본문에서 일반명사와 고유명사만을 추출하였다. 이후 도메인 특화 불용어(stopwords) 사전을 구축하여 분석에 불필요한 노이즈 단어를 제거하고, 단어 빈도-역문서 빈도(Term Frequency-Inverse Document Frequency) 알고리즘을 적용하여 특정 기사에만 과도하게 등장하거나 코퍼스(corpus) 전반에 걸쳐 변별력이 없는 단어를 필터링함으로써 정교한 어휘 사전을 구축하였다.

정제된 텍스트 데이터의 맥락을 분석하기 위해 비지도 학습(unsupervised learning) 기반의 자연어 처리 기법인 LDA 알고리즘을 적용하였다. 이는 대규모 문서 집합 내에 잠재된 주요 토픽의 비중과 관련 키워드들의 확률 분포를 수학적으로 역산하여 추출하는 기법이다. 더 나아가 뉴스 기사를 생산하거나 주도하는 발화자(수행 주체)에 따른 시각 차이를 규명하기 위해 기사의 내용과 주체를 기준으로 전체 데이터를 세 가지 그룹으로 계층화하여 교차 분석을 수행하였다. 세 개의 수행 주체를 보건기관, 커뮤니티, 기타로 구분하였다. 보건기관에는 보건복지부, 질병관리청, 보건소‧보건지소, 의료원, 국민건강보험공단, 치매안심센터 등을 포함하였고, 커뮤니티에는 주민센터‧행정복지센터, 경로당, 주민자치조직, 복지관과 마을회관 등을 포함하였다. 대학, 연구소, 학회, 기업과 민간단체 등은 기타로 분류하였다.

## 결 과

### 1. 진주시 노인의 노쇠에 대한 주관적 체감과 인식 구조

초점 집단 면담 자료를 주제 분석한 결과, 진주시 노인들이 체감하는 노쇠의 본질과 건강에 대한 인식은 총 10개의 핵심 주제와 구체적인 하위 주제로 도출되었다(표 1). 인터뷰에 참여한 노인들은 노쇠를 근감소증이나 특정 질환과 같은 단편적 상태로 규정하지 않고, 신체, 인지, 정신, 사회성이 유기적으로 얽혀 무너지는 ‘복합적이고 연쇄적인 기능 붕괴 현상’으로 인식하고 있었다.

결과에서 가장 주목해야 할 지점은 노인들이 ‘사회적 고립(social isolation)’을 노쇠의 비극적 결과일 뿐만 아니라 노쇠를 가장 먼저 촉발하는 선행 원인(trigger)으로 인식하고 있다는 사실이다. 참여자들은 “마음이 먼저 늙고 사람을 피하게 되면 몸도 금방 늙는다,” “집에만 있는 것이 병을 부른다”라며 심리‧사회적 위축이 신체적 근감소증보다 우선하여 나타남을 지적하였다.

이는 노쇠를 평가하는 임상 지표인 K-FRAIL (피로[Fatigue], 저항력[Resistance], 보행속도[Ambulation], 질병[Illness], 체중 감소[Loss of weight])의 신체적 측정치에 가려져 있던 ‘우울’과 ‘고립’이라는 내적 변수가 실제 노인의 삶에서는 훨씬 더 강력하게 작용할 수 있음을 시사한다. 아울러 노인들은 삶의 자립성을 상실하여 ‘가족에게 짐이 되고 요양병원에 갇히는 것’을 죽음이나 빈곤보다 더 큰 공포로 여겼으며, 이를 회피하기 위해 노인 일자리나 경로당의 공동 식사 등 다수가 어울릴 수 있는 사회적 연결망의 제공을 지역사회에 강하게 요구하였다.

### 2. 국내 언론 보도의 노쇠 담론 구조 및 주체별 시각차

전체 369건의 언론 기사에 대한 LDA 토픽 모델링 결과, 총 6개의 주요 담론이 추출되었다(표 2). 첫 번째 토픽(T1)은 운동, 근육, 단백질, 섭취, 근감소증 등의 단어를 중심으로 ‘노년기 근감소증 예방 및 영양‧운동 관리’로 해석하였다. 두 번째 토픽(T2)은 돌봄, 구강, 의료, 통합, 치과, 요양과 방문이 높은 가중치를 보여 ‘지역사회 통합돌봄 및 방문 의료‧구강 서비스’로 명명하였다. 세 번째 토픽(T3)은 노화, 나이, 질환, 당뇨병, 혈당, 스트레스와 생활습관을 중심으로 노화 속도와 만성질환 관리를 다루었다. 네 번째 토픽(T4)은 식사, 혈압, 혼밥, 혼자, 우울감 및 신체기능 저하가 함께 나타나 독거‧혼밥 노인의 신체‧정신건강 위험을 반영하였다. 다섯 번째 토픽(T5)은 기능, 평가, 개발, 기술, 임상, 프로그램과 측정으로 구성되어 신체기능 평가와 임상‧기술 기반 중재를 나타냈다. 여섯 번째 토픽(T6)은 노화, 예방, 사업, 보건, 코호트, 시범과 지역이 결합되어 지자체‧보건소 중심의 노화 예방 및 코호트 사업으로 해석하였다.

언론 보도 주체는 보건기관, 커뮤니티, 그리고 기타로 분류하였다. 보건기관 관련 기사에서는 노년기 기능 저하와 근육‧영양 관리, 지자체‧보건소 중심 만성질환 예방사업, 방문 의료‧구강 케어와 통합돌봄 체계가 주요 주제로 나타났다. 커뮤니티 관련 기사에서는 일상 밀착형 돌봄인 경로당 공동 식사, 독거노인의 식생활 지원, 구강‧영양 방문 돌봄, 마을 중심 통합돌봄 네트워크가 강조되었다. 기타 기사에서는 근감소증과 구강 쇠약의 임상적 위험, 세포 노화와 혼밥 등 생활양식, 디지털 헬스케어 및 고령자 평가기술이 주요 주제였다. 구강건강, 통합돌봄, 신체기능, 영양‧식사는 세 수행 주체가 공통으로 다루었으나, 보건기관은 제도와 사업, 커뮤니티는 일상생활 지원, 기타는 기전‧평가‧기술의 관점에서 접근하였다.

뉴스와 노인 인식의 두 자료는 신체기능과 이동성, 운동과 영양, 독거‧혼밥 및 사회적 고립, 지역사회 기반 예방과 돌봄의 중요성에서 수렴하였다(표 3). 특히 뉴스의 ‘독거‧혼밥 건강위험’ 토픽은 면담에서 외로움-활동 감소-우울-노쇠로 이어지는 악순환 인식과 연결되었다. 또한 보건기관과 커뮤니티 기사의 예방‧통합돌봄 주제는 노인이 요구한 공동 식사, 운동, 주민모임, 사회참여 지원과 같은 방향을 보였다.

반면 뉴스는 구강건강, 만성질환, 신체기능 측정, 코호트와 디지털 헬스케어 등 전문가‧제도‧기술 중심 요소를 확장하여 제시하였다. 노인은 뉴스에서 상대적으로 덜 드러난 자립성, 의욕과 자신감, 인지기능 변화, 가족에게 부담이 될 수 있다는 두려움을 강조하였다. 즉, 언론 담론이 노쇠를 측정하고 관리해야 할 위험상태로 설명했다면, 노인은 일상적 역할과 관계를 잃어가는 경험으로 설명하는 경향을 보였다.

## 논 의

이 연구는 노쇠에 관한 국내 뉴스 담론과 지역사회 노인의 주관적 인식을 함께 분석하여 공통점과 간극을 확인하였다. 뉴스에서는 근감소증, 운동과 단백질, 통합돌봄, 구강건강, 기능평가와 기술이 주요 의제로 형성되어 있었다. 노인 역시 움직임과 근력, 운동‧영양을 중요하게 보았으나, 건강의 기준을 자립적 생활과 사회적 관계 유지에 두었으며 노쇠를 신체‧인지‧정서‧사회 기능이 서로 영향을 미치며 악화되는 과정으로 이해하였다. 이는 건강 노화가 개인의 내재 역량과 환경의 상호작용을 통해 기능적 능력을 유지하는 과정이라는 세계보건기구의 관점과 부합한다[1].

노인의 인식에서 보행과 외부활동은 노쇠를 판단하는 구체적이고 관찰 가능한 신호였다. 이는 느린 보행과 근력 저하가 신체적 노쇠의 핵심 구성요소라는 기존 개념과 일치한다[2,3]. 그러나 면담 참여자들은 보행 저하를 단순한 신체 문제로 보지 않고 자신감 저하, 외출 감소와 관계 단절로 이어지는 출발점으로 설명하였다. 따라서 대국민 홍보에서는 ‘근육이 줄어드는 상태’만 강조하기보다 ‘걷는 속도가 느려지고, 하던 일을 줄이고, 사람을 만나는 일이 감소하는 변화’를 함께 제시하는 것이 노인의 경험에 더 부합할 수 있다.

외로움과 사회적 고립은 뉴스와 면담에서 공통으로 중요한 문제였다. 종단연구에서는 외로움이 이후 신체적 노쇠로 진행할 위험과 관련될 수 있음이 보고되었다[7]. 본 면담에서도 외로움은 활동 감소와 우울을 거쳐 노쇠를 촉진하고, 기능 저하는 다시 사람을 만나기 어렵게 하는 양방향 관계로 인식되었다. 지역사회 노쇠 예방사업은 운동 교실만으로 구성하기보다 소규모 모임 활성화, 친구 또는 건강 리더 연결 등 사회적 요소를 포함할 필요가 있다.

노인은 노쇠를 두려운 말로 받아들이면서도 자신의 노력으로 지연할 수 있다고 보았다. 국외 질적 연구에서도 노쇠에 대한 노인의 이해가 의학적 정의와 다르고, 의존성‧낙인과 연결될 수 있어 설명 방식과 용어 선택이 중요하다고 보고하였다[4,5]. 예방 메시지는 ‘나이가 들면 누구나 생기는 쇠약’이라는 숙명론을 피하고, 정상 노화와 구분되는 조기 신호, 개선 가능한 요인과 실제 참여 가능한 지역 서비스를 함께 알려야 한다. 운동은 전노쇠‧노쇠 노인의 근력, 보행, 균형과 신체수행을 개선할 수 있으며[8], 운동과 영양을 함께 제공하는 중재도 노쇠 관련 지표 개선에 도움이 될 가능성이 있다[9].

뉴스 수행 주체별 차이는 지역사회 전달체계 설계에 시사점을 준다. 보건기관은 예방사업과 통합돌봄의 제도적 기반을, 커뮤니티는 경로당 공동 식사와 일상적 돌봄을, 학술‧기술 부문은 평가도구와 디지털 기술을 제공하는 역할로 나타났다. 노인이 요구한 것은 이러한 요소를 별개의 사업으로 접하는 것이 아니라 생활권 안에서 연결된 서비스로 경험하는 것이었다. 보건소는 간편한 노쇠 선별과 기능평가를 실시하고, 결과에 따라 운동‧영양‧구강‧인지‧정신건강‧사회참여 서비스를 지역사회 복지사업과 연계하는 허브 역할을 수행할 수 있다.

본 연구에는 제한점이 있다. 첫째, 구글 뉴스 RSS 기반 자료는 검색 알고리즘과 언론사 본문 접근성의 영향을 받으므로 국내 전체 보도를 대표하지 않을 수 있다. 둘째, 초점 집단 면담은 일개 도심지역의 노인 9명을 대상으로 하여 다양한 노인 집단으로 일반화하기 어려우며, 모두 부부 세대 노인이어서 독거와 같은 사회적 문제에 대한 의견이 제한적일 수 있다. 그럼에도 본 연구는 대규모 뉴스 담론과 노인의 생활세계 관점을 통합하여 노쇠 커뮤니케이션과 지역사회 사업이 함께 다루어야 할 핵심 영역을 제시하였다는 의의가 있다.

결론적으로 국내 노쇠 관련 뉴스와 노인의 인식은 신체기능, 운동‧영양, 사회적 고립과 지역사회 예방의 중요성에서 일치하였다. 뉴스는 제도와 기술을 강조한 반면, 노인들은 신체(자립성의 중요), 인지, 사회활동 등 개인 단위의 건강 상태와 가족의 돌봄 부담 증가에 대한 걱정 등을 더 강조하였다. 따라서 공중보건 메시지는 노쇠를 정상 노화와 구별되며, 다차원적(신체, 영양, 사회활동 등)으로 예방하고 개선할 수 있는 개인의 건강 상태로 설명하고, 지역사회 사업은 기능평가와 함께 운동‧영양‧구강‧정신건강‧사회적 연결을 통합해야 한다(그림 1).

## Figures and Tables

**그림 1:** 언론 담론과 노인의 노쇠 인식: 지역사회 노쇠 예방을 위한 실천적 함의

**표 1:** 노인 초점 집단 면담을 통한 노쇠 관련 주제 도출 결과

핵심 주제(theme)	하위 주제(sub-theme) 및 주요 내용
1. 건강한 노화는 질병 부재가 아닌, 스스로 움직이며 함께 사는 삶	자립적 활동과 사회적 관계 유지가 건강한 노화의 핵심이며, 고립은 건강하지 않은 상태로 인식
2. 노쇠는 단순한 노화가 아닌, 기능과 의욕이 함께 무너지는 과정	보행‧근력‧피로 등 신체기능 저하와 의욕‧자신감 감소가 함께 나타나는 상태
3. 노쇠를 가르는 경계선은 ‘이동’과 ‘사회참여’	보행능력, 외출, 사람과의 만남 감소가 노쇠를 판단하는 중요한 기준
4. 노쇠는 신체 저하와 인지 저하의 동시다발적 경험	신체활동의 어려움과 함께 기억력‧길 찾기 등 인지기능 저하를 경험
5. 노쇠는 신체와 정신이 상호작용하는 악순환	신체기능 저하 → 의욕‧자신감 감소 → 활동‧사회참여 감소 → 추가적인 기능 저하
6. 사회적 고립은 노쇠를 촉진하는 중요한 요인	외로움, 대화와 인간관계 감소, 집에 머무는 생활이 노쇠를 가속한다고 인식
7. 노쇠는 개인의 노력으로 지연할 수 있음	걷기‧운동, 적절한 영양, 긍정적 태도와 지속적인 활동을 통해 노쇠 진행을 늦출 수 있다고 인식
8. 노쇠의 주요 인자는 외로움과 건강하지 않은 생활습관	외로움, 운동‧영양 부족, 만성질환, 수면 부족 등이 주요 원인으로 인식
9. 가장 큰 두려움은 가족에게 돌봄 부담이 되는 것	치매, 자립성 상실과 요양 의존으로 가족에게 부담을 주는 상황을 가장 크게 우려
10. 노쇠 예방은 지역사회 공동체 안에서 이루어져야 함	일자리, 공동 식사, 단체운동 및 주민모임 등 사회참여 기회와 지역사회 인프라를 요함

**표 2:** 뉴스 기사 자료에서 도출된 주요 담론 및 뉴스 주체별 주제

구분	주제	대표 핵심어	해석
T1	노년기 근감소증 예방 및 영양‧운동 관리	운동, 근육, 단백질, 섭취, 근감소증	근육 및 신체기능 저하 예방을 위한 운동‧영양
T2	지역사회 통합돌봄 및 방문 의료‧ 구강 서비스	돌봄, 구강, 의료, 통합, 요양, 방문	방문 진료‧구강 관리‧요양을 연계한 통합돌봄
T3	노화 속도 및 만성질환 생활습관 관리	노화, 나이, 당뇨병, 혈당, 스트레스	만성질환과 생활습관을 통한 노화 속도 관리
T4	독거‧혼밥 노인의 건강위험	식사, 혈압, 혼밥, 혼자, 우울감	사회적 고립과 신체‧정신건강 위험
T5	신체기능 평가 및 기술 기반 중재	기능, 평가, 개발, 기술, 임상, 측정	평가도구와 임상‧디지털 중재
T6	보건소 중심 예방 및 코호트 사업	노화, 예방, 사업, 보건, 코호트, 지역	지자체 시범사업과 모니터링 체계
보건기관	예방사업‧통합돌봄‧방문 의료	예방, 사업, 질환, 돌봄, 의료, 구강	제도적 공공보건 및 의료서비스
커뮤니티	공동 식사‧일상돌봄‧마을 네트워크	식사, 경로당, 혼자, 구강, 돌봄	생활권의 복지와 사회적 지원
기타	생물학적 노화‧근감소증‧디지털 평가	세포, 기능, 기술, 평가, 근감소증, 혈압	학술적 기전과 기술 개발

**표 3:** 뉴스 담론과 노인 인식의 통합적 비교

통합 영역	뉴스 담론	노인 면담	관계	공중보건 시사점
신체기능‧이동성	근감소증, 기능평가, 임상 중재	보행속도, 계단, 물건 들기, 외출	수렴	보행‧근력과 일상기능을 쉬운 언어로 설명
운동‧영양‧생활습관	단백질, 운동, 혈당‧당뇨 관리	걷기, 단백질, 규칙적 생활, 긍정적 태도	수렴	다영역 생활습관 중재와 반복 평가
사회적 고립	독거‧혼밥, 우울감과 건강위험	외로움-활동 감소-우울- 노쇠 악순환	수렴‧확장	공동 식사, 모임, 일자리와 사회적 처방 포함
지역사회 돌봄	보건소 사업, 방문 의료‧ 구강, 통합돌봄	운동프로그램, 주민모임, 사회참여 요구	수렴	보건소-복지기관-마을조직 연계
자립‧인지‧정서‧가족	상대적으로 제한적	자립성, 기억력, 의욕, 가족에게 짐이 될 두려움	노인 관점의 확장	인지‧정서 평가와 존엄‧자기결정 메시지 포함
기술‧제도	코호트, 측정기술, 디지털 헬스케어	구체적 기술보다 접근 가능한 대면서비스 강조	강조점 차이	기술은 관계와 서비스 접근을 보완하도록 설계
